# The need for harmonisation and innovation of neuropsychological assessment in neurodegenerative dementias in Europe: consensus document of the Joint Program for Neurodegenerative Diseases Working Group

**DOI:** 10.1186/s13195-017-0254-x

**Published:** 2017-04-17

**Authors:** Alberto Costa, Thomas Bak, Paolo Caffarra, Carlo Caltagirone, Mathieu Ceccaldi, Fabienne Collette, Sebastian Crutch, Sergio Della Sala, Jean François Démonet, Bruno Dubois, Emrah Duzel, Peter Nestor, Sokratis G. Papageorgiou, Eric Salmon, Sietske Sikkes, Pietro Tiraboschi, Wiesje M. van der Flier, Pieter Jelle Visser, Stefano F. Cappa

**Affiliations:** 1 Niccolò Cusano University, via Don Carlo Gnocchi, 3, Rome, Italy; 2 IRCCS Fondazione Santa Lucia, via Ardeatina 354, Rome, Italy; 3 University of Edinburgh, 7 George Square, EH8 9JZ Edinburgh, Scotland UK; 4 University of Parma, Via Gramsci, 14, Parma, Italy; 5 Medicina dei sistemi, Tor Vergata University, Via Montpellier, 1, Rome, Italy; 6 University Hospital La Timone, 264 Rue Saint-Pierre, Marseille, France; 7 Aix Marseille University, Jardin du Pharo, 58 Boulevard Charles Livon, Marseille, France; 8 National Fund for Scientific Research (F.R.S-FNRS), Quartier Agora place des Orateurs 1, Liège, Belgium; 9 Cyclotron Research Centre, University of Liege, Allée du VI août, 8, Liège, Belgium; 10 Dementia Research Centre, UCL Institute of Neurology, University College of London, Queen Square, WC1N 3BG London, UK; 11 Leenaards Memory Centre CHUV, Lausanne University Hospital, Rue du Bugnon 46, Lausanne, Switzerland; 12 Institut de la Mémoire et de la Maladie d’Alzheimer (IMMA), Hôpital de la Pitié-Salpêtrière, 47-83 Boulevard de l’Hôpital, Paris, France; 13 German Center for Neurodegenerative Diseases (DZNE), Holbeinstraße 13-15, Bonn, Germany; 14 Medical School, National and Kapodistrian University of Athens, Rimini street, 124 62 Haidari, Athens, Greece; 15 University Hospital of Liege, Liege, Belgium; 16 Alzheimer Center/dpt Neurology, VU University Medical Center of Amsterdam, Amsterdam Neuroscience, De Boelelaan 1118, Amsterdam, The Netherlands; 17 Fondazione IRCCS Istituto Neurologico Carlo Besta, Via Giovanni Celoria, 11, Milan, Italy; 18 Maastricht University Medical Centre, Dr. Tanslaan 12, Maastricht, The Netherlands; 19 IUSS Pavia, Piazza della Vittoria 15, 27100 Pavia, Italy; 20 IRCCS Centro San Giovanni di Dio, via Pilastroni 4, Brescia, Italy

**Keywords:** Neurodegenerative dementia, Cognitive assessment, Behavioural assessment, Neuropsychological tests, Longitudinal studies, European research

## Abstract

Cognitive, behavioural, and functional assessment is crucial in longitudinal studies of neurodegenerative dementias (NDD). Central issues, such as the definition of the study population (asymptomatic, at risk, or individuals with dementia), the detection of change/decline, and the assessment of relevant outcomes depend on quantitative measures of cognitive, behavioural, and functional status.

Currently, we are far from having available reliable protocols and tools for the assessment of dementias in Europe. The main problems are the heterogeneity of the tools used across different European countries, the lack of standardisation of administration and scoring methods across centres, and the limited information available about the psychometric properties of many tests currently in widespread use. This situation makes it hard to compare results across studies carried out in different centres, thus hampering research progress, in particular towards the contribution to a “big data” common data set.

We present here the results of a project funded by the Joint Program for Neurodegenerative Diseases (JPND) and by the Italian Ministry of Health. The project aimed at providing a consensus framework for the harmonisation of assessment tools to be applied to research in neurodegenerative disorders affecting cognition across Europe. A panel of European experts reviewed the current methods of neuropsychological assessment, identified pending issues, and made recommendations for the harmonisation of neuropsychological assessment of neurodegenerative dementias in Europe.

A consensus was achieved on the general recommendations to be followed in developing procedures and tools for neuropsychological assessment, with the aim of harmonising tools and procedures to achieve more reliable data on the cognitive-behavioural examination. The results of this study should be considered as a first step to enhancing a common view and practise on NDD assessment across European countries.

## Background

The large-scale availability of cohorts at the European level is a prerequisite for the construction of platforms for prevention and intervention studies in neurodegenerative dementias (NDD). A necessary step for the successful linking of existing or planned cohort studies is the harmonisation of clinical and biomarker data. In particular, an urgent need is to develop a consensus on methodologies to define and measure cognitive, behavioural, and functional status in target populations. The role of cognitive, behavioural, and functional assessment is crucial in longitudinal studies for several central purposes. A quantitative assessment of cognitive, behavioural, and functional status is needed for the definition of the study population as asymptomatic (individuals who present risk factors, such as ageing, but do not manifest cognitive symptoms or signs), at risk (individuals with mild cognitive impairment or subtle cognitive changes not fulfilling the criteria for dementia), or individuals with dementia (i.e. fulfilling the criteria for dementia according to a standard definition such as cognitive impairment impacting on social function and activities of daily living). In addition, the same measures are used for the detection of change/decline and for the assessment of relevant outcomes of interventions.

Currently, we are far from having available reliable protocols and tools for the assessment of dementias in Europe (for a recent US perspective on this issue, see [1]). The tools used are heterogeneous across different European countries, within an individual country, and even across different clinical and research institutes. This situation makes it hard to compare results across studies carried out in different centres, thus hampering research progress, in particular towards the contribution to a “big data” common data set.

The requirement for harmonised assessment of cognitive, behavioural, and functional symptoms has been extensively discussed in the field of dementia research [2]. Harmonisation recommendations have been proposed in the case of vascular cognitive impairment [3]. In the case of NDD, surveys of assessment tools for the most common NDD, Alzheimer’s disease (AD), used across Europe have been published by the European AD consortium [4] and by a task force of the European Federation of Neurological Societies [5], demonstrating a wide variety in assessment tools. In the last few years, the development of new diagnostic criteria for AD [6, 7], posterior cortical atrophy [8], frontotemporal dementia - behavioural variant [9], and primary progressive aphasia [10] all include a consideration of recommended, disease-specific testing procedures aiming at early diagnosis—although precise details of specific tests are often lacking, meaning that test selection is open to interpretation and, therefore, to potential inconsistency.

We present the results of a Working Group (WG) supported by the Joint Program for Neurodegenerative Diseases (JPND) and by the Italian Ministry of Health. The aims of the WG were: 1) to provide a set of practical recommendations for effective harmonisation procedures allowing us to optimise the utilisation of cognitive, behavioural, and functional data from on-going longitudinal studies; and 2) to identify areas in which further research is needed in order to propose harmonised innovative assessments maximising the effectiveness of deep phenotyping, considering in particular the possible translational impact of cognitive neuroscience research in areas such as memory, language, spatial orientation, action organisation, and social cognition.

This paper is an extended version of the final report on the JPND website (http://www.neurodegenerationresearch.eu/initiatives/jpnd-alignment-actions/longitudinal-cohorts/call-for-working-groups/call-results). It is structured in two main sections. In the first section, we describe the methods. In the second section, we present the results, in separate sub-sections for each phenomenological dimension we reviewed, including both: 1) an analytical discussion of current evidence; and 2) recommendations for further developments of practice and research.

## Methods

**Fig. 1:**
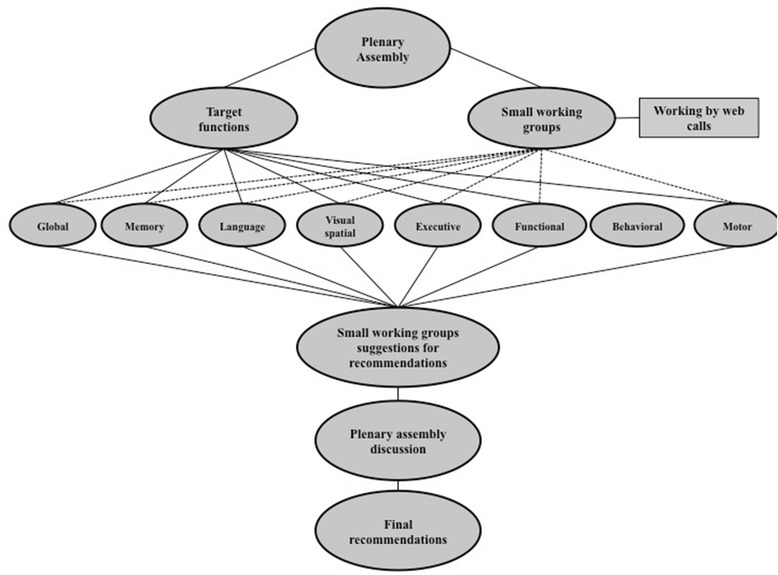
The flow chart that synthetises the main steps of the project

The flow chart shown in Fig. 1 synthetises the main steps of the project. The project was selected in a competitive call by the Joint Program for Neurodegenerative Diseases for “Working Groups to Inform Cohort Studies in Neurodegenerative Disease Research”. The recruitment of the group members was the responsibility of the proponent, who, during the pre-submission phase, contacted researchers (neurologists and neuropsychologists) with a specific competence in the diagnosis and psychometric assessment of dementia, as documented by their scientific publications in peer-reviewed international journals. Additional criteria were expertise in specific sub-fields of assessment, representation of main European research centres involved in longitudinal studies of NDD, and availability to participate in the working group programme (opening meeting, 6 months of at-distance work, final meeting). A total of 22 experts from eight European Union countries (Belgium, Denmark, France, Germany, Greece, Italy, the Netherlands, and the UK) and from Switzerland accepted the invitation to participate.

In a first workshop participants discussed the general organisation of the workplan: reviewing the available evidence on neuropsychological assessment of NDD in Europe. A general distinction was made between “global assessment”, referring to procedures used for the initial screening of the patient’s general cognitive status, and “detailed assessment”, encompassing target phenotypic dimensions of dementias, intended as the main phenomenological changes of NDD involving cognitive, behavioural, motor, and functional domains that are measurable by the administration of psychometric tools. These target dimensions were defined with plenary consensus and were identified taking into account both the clinical manifestations that are common to all NDD and signs that are specific to the early stages of the different syndromes. The following target dimensions were, thus, defined as follows: episodic memory (early involvement in the typical form of AD), language (early impairment in primary progressive aphasias), executive functions (early involvement in frontotemporal dementias), visual-spatial abilities (early impairment in posterior cortical atrophy and dementia with Lewy bodies), behavioural symptoms (early involvement in frontotemporal dementia – behavioural variant), motor (early involvement in corticobasal degeneration and in dementias with extrapyramidal symptoms), and functional status (common to the early phases of all NDD).

Small groups encompassing three to four researchers were then created, each dedicated to one target dimension. The task of the small groups was to review current methods used to investigate target phenotypic dimensions of dementia, identify critical issues, and provide a definite set of operational recommendations for harmonisation purposes: identifying un-met needs for each specific domain, taking into account, in particular, the need for adequate information about psychometric qualities (i.e. validity, reliability, sensitivity, specificity). Critical issues in each area were identified based on existing evidence as well as the participants’ knowledge and clinical expertise.

The initial indications proposed by these small groups were then discussed in web-based conferences involving the whole consortium. In these conferences, statements, procedures, and indications by the small groups were revised.

A consensus on the available evidence and un-met needs for each cognitive domain, identified as critical in the assessment and monitoring of NDD, was achieved in a final workshop, involving the entire consortium, either by physical participation or web-conference. The consensus on each specific issue was achieved with the agreement of all participants.

## Results

### Global assessment

#### Discussion of current evidence

The Mini Mental State Examination (MMSE) is currently the most widely used test for cognitive screening in clinical practice [11], and is mentioned by several guidelines for the assessment of dementia and cognitive disorders. Indeed, it shows good sensitivity and reliability with Cronbach’s α = 0.91 [12]. Moreover, some MMSE items (i.e. intersecting-pentagon copy) were found to reliably discriminate between AD and dementia with Lewy bodies (DLB) [13] in an autopsy-verified population. Nevertheless, the MMSE suffers from some important limitations. It shows poor specificity for people with a low educational level, who may achieve low scores although healthy, and poor sensitivity for people with a high educational level who can obtain high scores even when impaired; it is highly sensitive to memory and language disorders but not to executive functioning [14].

The Montreal Cognitive Assessment (MoCA) is another brief screening measure for cognitive disorders [15] consisting of a 30-point test administered in 10 min, focusing on memory, visual-spatial, executive, and language function, and orientation to time and place. The MoCA has the advantage of being short and easy to administer with a good sensitivity (86%) for mild cognitive impairment (MCI) and AD (97%) and for the post-stroke cognitive screening [16]. It is easily available via a dedicated website providing free test forms in a number of languages, including parallel versions.

The Frontal Assessment Battery (FAB) [17] includes six sub-tests exploring conceptualisation, mental flexibility, motor programming, inhibitory control, and environmental autonomy, and is considered a reliable tool to investigate dysexecutive symptoms in frontal-related disorders. However, its usefulness to discriminate between the dysexecutive profiles of different NDD is controversial [18, 19]. In particular, the results of a study involving individuals with mild AD and mild frontotemporal dementia (FTD) documented that only the FAB sub-test “go/no-go” detected significant between-group differences, whereas performance on the other sub-tests was quite similar. However, the authors found good concurrent validity (i.e. significant correlation between FAB and individual executive test scores).

Test batteries such as the AD Assessment Scale-Cognition (ADAS-CoG) [20], Cambridge Cognitive Assessment-Revised (CAMCOG-R) [21], and Addenbrooke’s Cognitive Examination (ACE-R) [22] have the advantage of parallel forms but the assessment of executive functions is limited. ACE-R takes between 12 and 20 min (average 16 min) to administer (and score) and examines attention/orientation, memory, fluency, language, and visual-spatial functions. Future work is needed to establish the efficacy of a brief battery such as ACE-R in different types of dementia, even though it has been indicated to be effective in differential diagnosis of different forms of Parkinsonism [23].

The Mattis Dementia Rating Scale (MDRS) [24] is a battery that assesses attention, perseveration, construction ability, conceptualisation, and memory, and was recently recommended by the Movement Disorder Society for the assessment of dementia in individuals with extrapyramidal disorders [25]. However, administration may be considered too lengthy for a first global screening test.

#### Recommendations

The panel recommended a step-wise approach to be followed in the assessment of a person in whom dementia is suspected. As a first step, a short screening should be performed (less than 15 min for administration). The MMSE, MoCA, Mini-CoG [26], ACE or ACE-R, and the FAB are recommended for this purpose. In the case of clinical and psychometric evidence suggesting a cognitive impairment, a second-level examination should be performed. At this level, an examination by a specialist should confirm/verify the initial diagnostic hypotheses. For this purpose, the following tests could be used: ADAS-Cog, CAMCOG-R, and the MDRS.

The above two steps specifically refer to the examination of global cognitive functioning. The third step is the in-depth examination of the neuropsychological profile by means of tests specific to each cognitive domain.

### Detailed assessment area

In this section, we report results on the phenotypic dimensions of dementia defined by the consortium. Recommendations for the assessment of episodic memory, language, executive functions, visual-spatial abilities, behavioural symptoms, and motor and functional status are summarised in Table 1.

**Table 1:** Synoptically report of the recommendations for further developments of clinical research in the field of neurodegenerative dementias (NDD)

Target phenotypic dimensions	Open issues	Papers investigating psychometric properties of recommend tools	Recommendations	Psychometric properties
Episodic memory abilities	For each test proposed for clinical use we should know the sensitivity and specificity for AD and other NDD. Other issues to consider include: age effects, practice effects, ceiling and floor effects, repeatability, ease of administration, and correlation with biomarkers.	[30–35, 38]	Among extant tools, the FCRST and the Visual Short-Term Memory Binding Test are good candidates to discriminate between AD-related memory deficits and memory disorders occurring in other NDD.	Good specificity for AD
Language abilities	The extent of language assessment should be closely linked to the aim of the investigation. A consensus on a high-quality cross-language naming task is heavily needed. Lack of standardised tools to differentiate dementias that are not primarily characterised by language disorders	[41, 44]	In most settings, picture naming is the test of choice (e.g. Boston Naming Test). In investigations of progressive language disorders, a more comprehensive evaluation including an analysis of extended speech production as well as sentence-level tasks is recommended. Communication abilities should be assessed also as a key component of the functional profile	Picture naming tests are sensitive but not specific for dementia, as a naming disorder is a pervasive aspect of many NDD
Executive functions	Many tests to examine executive function of shifting, inhibition, and updating have unknown ecological validity. Few validated tools are currently available for the assessment of emotional processing and social cognition. There is no convergent agreement on the modular organisation of executive domain.	[62, 63, 68]	Executive functions: at a screening level at least two tests investigating two sub-components of the executive domain should be used. The following individual tests can be used: Stroop test, Trail Making Test, Wisconsin Card Sorting Test, Verbal Fluency, Emotional processes and social cognition: the Social and Emotional Assessment battery can be applied to assess emotion recognition and social cognition	Inhibition tests (e.g. Stroop test) are the most sensitive to AD. The Social and Emotional Assessment battery shows good sensitivity to FTD-behavioural symptoms In general, executive tests show low test–retest reliability and low ecological validity.
Visual-spatial abilities	A main issue is represented by the relatively low specificity of the available tools. Low specificity is attributable to the difficulty in differentiating between basic visual processes alterations and proper spatial disorders.	[81, 84, 86, 87]	Useful tools for the global assessment of visual-spatial abilities are the VOSP and the BORB batteries that allow the examination of multiple visual-spatial components. For a short screening, the Rey-Osterrieth figure and Benton Judgement of Line Orientation could be administered.	The VOSP has good sensitivity to AD. The contribution of Rey-Osterrieth complex figure test to differential diagnosis is controversial. The Benton Judgement of Line Orientation test can differentiate patients with DLB with psychotic symptoms from both patients with DLB with parkinsonian symptoms and patients with AD.
Behavioural symptoms	Available tools have low specificity hampering the possibility to differentiate between different NDD	[94, 95, 97]	The choice of the best tool to assess BPSD in dementia should be guided by a syndromic approach. The Neuropsychiatric Inventory (NPI) is a reliable scale for the assessment of a wide range of BPSD in dementia.	The NPI has good specificity for DLB compared to AD. The Neuropsychiatric Inventory-Clinician rating scale (NPI-C) has good inter-rater reliability.
Motor symptoms	Lack of validated tools to assess motor symptoms specific to the different NDD	[103]	The use of the following disease-specific tools with good validity is recommended: UPDRS; UHDRS PSPRS; UMSARS; Clinical examination according to El Escorial criteria for ALS. For the assessment of ideomotor apraxia, the Dementia Apraxia Test (DATE) can be applied.	The DATE shows a good capacity to discriminate between individuals with AD and individuals with FTD-behavioural variant
Functional abilities	Some currently used IADL tools have relatively low evidence of validity. Lack of tools for the assessment of functional abilities in very early stages of dementia (i.e. mild cognitive impairment)	[113, 114, 116]	Although further research is needed to investigate quality aspects of IADL instruments, promising results have been found for several questionnaires, including the Everyday Cognition (ECog), the Cognitive Function Instrument (CFI), and the Amsterdam IADL questionnaire.	The Lawton IADL has good reliability. Some everyday cognition (ECog) sub-items (i.e. language sub-items) allow us to differentiate between MCI and dementia. The Amsterdam IADL questionnaire has good sensitivity to dementia-related changes over time

*AD* Alzheimer’s disease, *ALS* amyotrophic lateral sclerosis, *BORB* Birmingham Object Recognition Battery, *BPSD* Behavioural and Psychological Symptoms of Dementia, *DLB* dementia with Lewy bodies, *FCRST* Free and Cued Selective Reminding Test, *FTD* frontotemporal dementia, *IADL* instrumental activities of daily living, *MCI* mild cognitive impairment, *PSPRS* Progressive Supranuclear Palsy Rating Scale, *UHDRS* Unified Huntington’s Disease Rating Scale, *UMSARS* Unified Multiple System Atrophy Rating Scale, *UPDRS* Unified Parkinson’s Disease Rating Scale, *VOSP* Visual Object and Space Perception battery

#### Episodic memory functions

##### Discussion of current evidence

Episodic memory disorders are a key element of the cognitive decline that defines the clinical staging of AD. Memory disorders also occur in many other conditions, including depression and healthy ageing, leading to diagnostic uncertainty. Hence, memory tests are sensitive but may not be specific. Moreover, performance on such tests reaches a floor very early in the disease, making them a poor marker of disease severity and progression.

The most widely used episodic memory tests for the identification of the amnestic syndrome of AD are based on list learning and delayed recall. These tests include different versions of the paired-associate learning [27] and the Rey Auditory Verbal Learning tasks [28]. It has been proposed that tests that control for attention and effective encoding and that can facilitate retrieval are particularly suitable to identify an amnestic syndrome of the hippocampal type, typical of AD. Memory tests with cueing, either at the bedside (5-Word Test) [29] or administered by neuropsychologists (Free and Cued Selective Reminding Test (FCSRT)) [30], have shown good specificity for AD [31]. It should be taken into account that the above tools place heavy demands on verbal abilities. This significantly reduces their applicability in conditions characterised by severe language disorders (e.g. primary progressive aphasia (PPA)). Some neuropsychological tests with limited language demands have been proposed. Among these, the DMS48 [32] and the Visual Short-Term Memory Binding Test [33, 34], which tap long-term recognition memory and associative processes within short-term memory, respectively, showed good specificity for AD. They have the additional benefit of being unaffected by healthy ageing or low education levels. The recently proposed “5 Objects Test” has the advantage of being unaffected by low education levels and of having limited language demands (i.e. it requires locations of five everyday objects, immediately after placement, and after a brief period of time) [35].

A new area of research is represented by the study of prospective memory functioning and, in particular, by the potential contribution of prospective memory procedures to discriminate between different pathological conditions [36].

##### Recommendations

Additional information about the sensitivity, specificity, and positive and negative predictive value of each test proposed for clinical use in AD and in other dementias should be achieved [37, 38]. In this regard, it must be underlined that memory tests for dementia and earlier stages of impairment, such as MCI, have different requirements. The former must account for the distributed network dysfunction of manifest dementia, the latter need to be able to detect early and circumscribed deficits. Thus, the sensitivity of clinical tests is likely to benefit from incorporating the assessment of multiple memory processes, whereas sensitivity to early AD conditions is likely to benefit from process-specificity to the early stages of AD pathology involving sub-sectors of the medial temporal lobe. Other issues to consider include age effects, practice effects, ceiling and floor effects, repeatability, ease of administration, and correlation with biomarkers. Indeed, the distinction between neuropathological changes in healthy ageing and AD is disputed, making it challenging to define clear boundaries between the two.

Among extant tools, in order to discriminate between AD-related memory deficits and memory disorders occurring in other NDD, at the moment it is recommended to use more than one test. Among the most interesting candidates for high specificity in the very early stages of disease are the FCSRT and the Visual Short-Term Memory Binding Test.

#### Language abilities

##### Discussion of current evidence

Only a minority of tools have been developed to assess language impairments either in those NDD in which it is not a prominent feature (typical AD) or in NDD in which language disorders represent the main determinant of clinical presentation (PPA).

The most widely used naming test is the Boston Naming test [39, 40]. Originally developed for stroke aphasia, it has been adapted to wider applications with the development of several forms (60, 30, and 15 items). Extensive data are available about its sensitivity (individuals with AD vs. healthy controls). Two other tests currently used are the Graded Naming test [41] and the Aachener Aphasie Test (AAT) [42]. Important to evidence is that picture naming tasks are sensitive to AD [43] but they are less specific, as a naming disorder is a pervasive aspect of many NDD [44].

Word generation tests such as phonological and categorical fluency are widely used. However, due to the recruitment of various cognitive processes (e.g. working memory and executive processes) their specificity is relatively low [45]. The Pyramid and Palm Trees Test [46] assesses non-verbal semantics, and is highly specific for the semantic variant of PPA [47].

The assessment of sentence processing entails production and comprehension. To assess production abilities, the most used stimuli are the Cookie theft from the Boston Diagnostic Aphasia Examination (BDAE) and the picnic picture from the Western Aphasia Battery (WAB). A scoring system for PPA is also available [48]. Phrase construction tests have been proposed as a faster, quantitative way to assess production abilities (Northwestern Anagram Test (NAT)) [49].

The most widely used tool to assess sentence comprehension is the Token Test, originally developed as a sensitive test for auditory comprehension in stroke aphasia [50]. The Test for the reception of grammar (TROG) [51] or the Cycle-R [52] have been successfully applied in neurodegenerative diseases to identify specific grammatical deficits.

The assessment of language disorders should also include the evaluation of reading and spelling abilities. In this case, The National Adult Reading Test (NART) [53], or its adaptation into other languages, is often used and also represents a valid estimation of premorbid IQ in dementia. However, as the NART relies on the ability to read irregular words, its application in languages with regular orthography (e.g. Italian and Spanish) is problematic.

Only recently there has been an interest in the development of specific language batteries for NDD. These include the Cambridge Semantic Memory Test [54] and the Sydney Language Battery (SYDBAT) [55]. The latter two, however, concentrate on semantic processing rather than an attempt to cover all aspects of language. Furthermore, the Amsterdam-Nijmegen Everyday Language Test (ANELT) [56] is an example of a test focusing on communicative ability rather than grammatical competence. The clinical use of extensive language examinations is required only in the case of PPA.

##### Recommendations

The extent of language assessment should be closely linked to the aim of the investigation. In general, lexical semantic processes are more affected than syntactic or phonological processes in the early phase of most NDD. In most settings, picture naming is the test of choice. While many tests are in use, information about psychometric properties, as well as parallel and multi-lingual versions, is needed. In investigations of progressive language disorders, a more comprehensive evaluation including an analysis of extended speech production as well as sentence-level tasks is recommended. Indeed, impairment at the sentence level can occur also in individuals with intact single-word processing; accordingly, in this case, any language assessment not including sentence processing is incomplete and potentially misleading.

#### Executive functions

##### Discussion of current evidence

Executive disorders are reported in the early stages of different dementia syndromes, and their role in differential diagnosis is a matter of debate. Indeed, under the umbrella term of “executive functions” are included several mental operations that are partly differentiable. Accordingly, different models of the executive domain have been proposed [57]. This highlights one main difficulty in identifying suitable tests for assessment. In this regard, reduced performance on tests sensitive to controlled inhibition, set-shifting and updating, is mainly associated with dorsolateral frontal dysfunction, whereas performance on others tasks sensitive to emotional/social cognition is mainly associated with frontotemporal dysfunction [58].

As for tests sensitive to controlled inhibition, set-shifting and working memory, Stopford et al. [59] showed that they allowed detecting qualitatively distinct profiles between individuals with FTD and with AD. In fact, impairments in individuals with FTD reflected deficits in attention, set shifting, and response inhibition, while impairments in individuals with AD were influenced by information load and working memory capacity.

Among these tests, inhibition tests are the most sensitive to AD and, more particularly, controlled inhibition (as, for example, in the Stroop or Hayling tasks) [60]. However, these tasks do not allow us to easily distinguish between AD and the FTD-behavioural variant on a quantitative basis [61]; probably a better way to distinguish the two populations is to use qualitative measures [62]. Indeed, most of the clinical or conventional tests of executive functions may be limited by their low test/re-test reliability and by reduced ecological validity [63].

The FAB [17] and the Institute of Cognitive Neurology (INECO) frontal screening battery [64] are commonly used tools for the screening of these executive functions. However, the differential weight of their sub-items remains debatable.

Another applied approach to capture the variety of executive functions is to combine different tests, and look for the number of failed tests to assess executive dysfunction. The GREFEX battery [65] includes cognitive and behavioural tools that are standardised, validated, and normalised for a French-speaking population. The battery is composed of a questionnaire (Behavioural Dysexecutive Syndrome Inventory; Inventaire du Syndrome Dysexécutif Comportemental (ISDC)) and seven cognitive tasks (Stroop task, modified Six Elements Test, Trail-Making test, Adapted version of the Brixton task, a dual-task test, verbal fluency, and modified card sorting test). A similar battery is the Behavioural Assessment of Dysexecutive Syndrome (BADS) [66] that includes sub-tests exploring the different dimensions of the executive domain.

Few tools are currently available to assess emotional processes and social cognition. A classical test investigating emotional recognition was developed by Ekman et al. [67] in which subjects have to identify basic emotions from facial expression of unknown people presented in photographs. The Iowa Gambling task is another popular task developed to assess decision making abilities in situations involving emotional activation [68].

False belief and Faux Pas tasks are one way to assess theory of mind (ToM), i.e. the ability to infer the mental state of somebody else. Although both Faux Pas and false belief tasks have been shown to detect a differential impairment profile in different NDD, in particular in the comparison of individuals with FTD-behavioural variant and individuals with AD [69], there is no consensus as to which test might be more useful for diagnosis and for differential diagnosis of dementia. Recently, some batteries sensitive to the phenomenological manifestations of FTD-behavioural variant have been proposed. Among these, the Social and Emotional Assessment battery (SEA) [70], assessing emotion recognition and ToM abilities, has been shown to be a promising tool for the early detection of FTD-behavioural symptoms.

##### Recommendations

Due to the multifaceted nature of the executive domain, the recommendation is to include tests assessing different executive subcomponents such as the Stroop test (selective attention and inhibition) [71], Trail Making Test (set-shifting) [72], Wisconsin Card Sorting Test (set-shifting and set-maintenance) [73], and verbal fluency (strategic access to lexicon) [74]. A comprehensive assessment of executive abilities can be achieved by the use of standardised tests batteries such as the BADS or the GREFEX battery.

Few standardised tests are currently available for the specific assessment of social cognition in dementias. Among these, promising data have been reported for the SEA.

#### Visual-spatial abilities

##### Discussion of current evidence

Dementia-related visual impairment is often neglected. However, visual impairments have a profound impact upon everyday life, with previous research demonstrating that spatial perception abilities are more strongly associated with activities of daily living than episodic and verbal short-term memory [75]. Visual impairment contributes to problems as diverse as falls and poor diet, as well as to challenging behaviours, hallucinations, and delusions.

One limitation in many evaluations of cortical visual function is that assessments are limited to higher order visuoperceptual (recognition and identification of objects, faces and scenes and their features) and visuospatial (localisation of objects and representation of the spatial relationship between oneself and objects in the environment [76]) processing without consideration of the integrity of fundamental, basic visual processes supported by striate and extrastriate occipital cortex (basic form, colour, motion, and location processing). Without testing basic visual functions, it is difficult to determine whether higher-order object and space-perception deficits are attributable to parietotemporal tissue loss, or in fact result from a more fundamental de-afferentation of these areas due to occipital lobe disease. Correct attribution of deficits may be particularly important in clinical practice to understand phenomenological heterogeneity in AD or to evaluate whether there are different parietal, occipitotemporal, and primary visual subtypes of PCA [77].

Conversely, there is a need to consider the impact of cortical visual dysfunction on other domains of cognitive test performance, particularly given the number of neuropsychological assessments with visually mediated instructions, stimulus presentation, or response formats. For instance, tests of episodic memory with explicit visual demands in encoding and/or retrieval (e.g. Rey-Osterrieth figure copy) are obviously unsuitable for individuals with prominent cortical visual impairment. Similarly, a majority of tests of executive functions are partly or wholly visually mediated. Less obvious are the more implicit visual demands of tests such as verbal paired associate learning that often draw on mental imagery [78]. The clinical experience in evaluating episodic memory function in individuals with PCA suggests that two alternate forced choice recognition memory tests [79] are particularly suitable (although not necessarily sensitive).

Visuoperceptual and/or visuospatial impairments are central to the diagnostic criteria of several dementias (DLB [80] and corticobasal degeneration (CBD) [81]). However, arguably the cardinal ‘visual dementia’ is posterior cortical atrophy (PCA), a clinical-radiological syndrome primarily characterised by impaired cortical visual function with relative preservation of episodic memory and insight [82].

Currently used batteries to detect deficits in early visual processing (e.g. figure-ground discrimination) and object and space perception include the Visual Object and Space Perception battery (VOSP) [83, 84] and the Birmingham Object Recognition Battery (BORB) [85]. For a short screening the Benton Judgement of Line Orientation [86, 87] and the Rey-Osterrieth figure [88–90] are commonly used.

##### Recommendations

The potential effect of basic visual deficits on performance should be taken into account for the choice of the tests to be used for clinical assessment of visual-spatial disorders. With this in mind, useful tools for the global assessment of visual-spatial abilities are represented by the VOSP and the BORB that allow the examination of the different visual-spatial components. For the purpose of a short screening, the Rey-Osterrieth figure and Benton Judgment of Line Orientation can be reliably administered.

In order to accurately choose the different sub-tests, it is quite important to take into consideration the patient’s comprehension capacity. Indeed, as discussed above, basic visual deficits could contribute to poor performance on tasks designed to evaluate higher order visual processes. This observation leads to the more general issue of test validity. For example, certain VOSP visuospatial sub-tests are better suited to individuals with moderate typical AD (instructions for the Number Location sub-test are more difficult to understand than those for the Dot Counting sub-test).

#### Behavioural symptoms

##### Discussion of current evidence

Neuropsychiatric symptoms (NPS), otherwise defined as Behavioural and Psychological Symptoms of Dementia (BPSD), are present in up to 90% of all dementias [91] and in 35–85% of MCI individuals [92]. They require assisted living or nursing facility placement and are highly clinically relevant since severe BPSD cause significant distress on caregivers, and affect quality of life and prognosis.

BPSD are heterogeneous and include the following: agitation, aberrant motor behaviour, anxiety, depression, elation, irritability, apathy, disinhibition, delusions, hallucinations, stereotypic behaviour, sleep or appetite changes, and abnormal sexual behaviour. The most frequent BPSD are apathy, depression, irritability, agitation, and anxiety. There is evidence of a stage-effect of BPSD in dementia. Affective symptoms such as depression, anxiety, and apathy would be prevalent in mild dementia. Psychotic signs such as delusions, hallucinations, elation, disturbances in motor function, and aberrant vocalisations, instead, would be more frequent in moderate to severe dementia. A recent study in a large population of individuals with AD reported five distinct neuropsychiatric syndromes. The apathetic syndrome (as a unique syndrome) was the most frequent, followed by affective syndrome (anxiety and depression), psychomotor (agitation, irritability, and aberrant motor behaviour), psychotic (delusions and hallucinations), and manic (disinhibition and euphoria) syndromes [93]. More than 75% of individuals with AD presented with one or more of the syndromes. The study supports a syndromic approach, and suggests that the clinicians need to incorporate a thorough psychiatric examination of individuals with AD. However, it is important to underline that around 50% of individuals with AD, FTD, vascular dementia (VaD), and DLB show at least four symptoms simultaneously. This makes it difficult to individualise a cluster of symptoms that may improve differential diagnosis accuracy.

The assessment of BPSD is performed by using questionnaires for patients and caregivers, focused on single or multiple signs. The Frontal Behavioural Inventory (FBI) [94] is an interesting tool for the purpose of differential diagnosis and to monitor the progression of individuals with prominent behavioural symptoms. For AD, the first behavioural rating scale was the BEHAVE-AD [95]. This scale investigates 25 behavioural symptoms and provides a rating of caregiver’s burden. Currently, one of the most widely used and reliable tools for the assessment of BPSD in dementia is the Neuropsychiatric Inventory (NPI) [96]. The NPI is a structured interview performed with the caregiver that assesses behavioural disorders belonging to 12 main categories (depression, apathy, agitation, etc.), rating their frequency, severity, and the caregiver’s distress. The NPI was shown to be sensitive to treatment effects and able to distinguish different NDD [97–99]. A revised version of the NPI was recently proposed, named the Neuropsychiatric Inventory-Clinician rating scale (NPI-C) [100].

##### Recommendations

The choice of the best tool to assess BPSD in dementia should be guided by a syndromic approach. Accordingly, all BPSD should be investigated by rating their severity and frequency across the different stages of dementia that include the very early phases. When needed for an in-depth assessment of individual behavioural dimensions, specific ad-hoc scales should be used. The NPI is a reliable tool for an extensive assessment of BPSD in dementias and, thus, it is recommended. However, for the assessment of individual affective dimensions more specific tools such as the Dimension Apathy Scale [101] and The Geriatric Depression Scale (GDS-15) [102] are recommended.

#### Motor symptoms

##### Discussion of current evidence

Across European countries, not all clinicians use motor scales in their practice with persons presenting cognitive-behavioural disorders. This point is well outlined by results of a recent survey (337 responses from 33 countries; presented as a poster at the European Association of Neurology meeting in Berlin on 20–23 June 2015 by Symonds and Bak) documenting that 25% of neurologists and 70% of psychiatrists do not assess motor functions regularly. Indeed, most clinicians underestimate the frequency of motor symptoms in memory clinics (according to the above-mentioned survey, most clinicians believe that only 0–20% of patients in the cognitive clinic have motor problems, while the literature points to a much higher frequency of 30–50%).

This is problematic, since in the so-called subcortical dementias such as, for instance, dementia associated to Parkinson’s disease (PDD), supranuclear palsy (PSP), CBD, Huntington disease (HD), and VaD, motor involvement is a marker. In some cases the onset of a cognitive-motor feature can be pathognomonic, e.g. ideomotor apraxia in CBD, although it can also occur in AD [103]. Systematic studies also show that motor signs are common even in dementia with main involvement of cortical brain areas such as AD [104] and PCA [105].

The only commonly used scale to motor symptoms is the Unified Parkinson’s Disease Rating Scale (UPDRS), which has been designed specifically for Parkinson’s disease (PD) and does not cover, therefore, many phenomena which can be of importance in individuals with dementia (e.g. amyotrophic features pointing to amyotrophic lateral sclerosis (ALS)). Unfortunately, a brief motor screening tool with standard norms and validity data that are not disease-specific and could be used in a wide range of diagnoses in a memory clinic is not available. Ideally, this tool should contain items investigating areas of motor function relevant to dementias: Parkinson symptoms (PD, DLB, PSP, CBD, and multiple system atrophy (MSA)); amyotrophic features (ALS/FTD); cerebellar features (MSA, alcoholism, spinocerebellar ataxea-17, etc.); and higher order motor disorders such as praxis, motor sequencing etc. (CBD, PCA). Recently, the Dementia Apraxia Test (DATE) [106] was proposed to evaluate ideomotor apraxia which has a sensitivity of 91% and specificity of 71%, with a good capacity to discriminate between AD and individuals with FTD-behavioural variant.

##### Recommendations

A global tool that allows for the screening of motor symptoms associated with different dementias is not available. The use of the following disease-specific tools that show a good validity level is recommended: UPDRS; Unified Huntington’s Disease Rating Scale (UHDRS); Progressive Supranuclear Palsy Rating Scale (PSPRS); Unified Multiple System Atrophy Rating Scale (UMSARS); and Clinical examination according to El Escorial criteria for ALS. The development of a brief, clinically applicable and easy-to-use non-disease-specific motor screening tool should be a matter of high priority. The recently developed Edinburgh Motor Assessment (EMAS) [107] could fill the gap, but it will require collection of normative data in patients and healthy controls of different ages and a validation against the above-mentioned established motor tests. Promising data for the assessment of ideomotor apraxia were reported for the DATE that could thus represent an interesting tool to be applied for future research.

#### Functional abilities

##### Discussion of current evidence

Impairments in instrumental activities of daily living (IADL) influence everyday lives of patients and caregivers, and are essential for the planning of care needs and for legal and financial matters. As such, functional assessment provides highly ecologically valid information. In clinical practice, an important objective of functional assessment is its ability to detect the impact of cognitive impairment on everyday functioning, as this is an important diagnostic criterion for dementia [6]. Additionally, the distinction between MCI and dementia is characterised by the absence of IADL impairments in the former, and presence of IADL impairments in the latter [108]. The assessment of everyday functioning is therefore crucial both in clinical practice and research studies. Several longitudinal studies have demonstrated that IADL measures in the healthy elderly are related to incident dementia, indicating that subtle impairment in IADL might occur earlier in the disease course [109]. These findings underline the relevance of a functional assessment for the early diagnosis of dementia [110]. IADL assessment is also relevant for the measurement of change over time. In particular, in clinical trials, IADL is an outcome measure required by the Food and Drug Administration to demonstrate clinically relevant changes [111].

There is an abundance of IADL instruments, ranging from self-report questionnaires and informant-based questionnaires to performance-based instruments [112]. Different measurement methods serve different purposes [113], with questionnaires completed by the informant or study partner being the most common assessment method. When focusing on validity there are large differences in the content between different instruments. Instruments combining IADL and basic activities of daily living (BADL) into a single instrument are less sensitive to cognitive impairments and this might lead to an under-estimation of functional impairment [113]. Within the measurement of IADL, instruments also vary as to whether they focus on cognitive aspects of each activity or target IADL performance. In a systematic review, in which quality aspects and psychometric properties of IADL instruments were rated on a predefined scale, it was demonstrated that IADL instruments lacked relevant information on face validity, reliability, content, and construct validity [112]. Other systematic reviews supported this notion that psychometric aspects and quality aspects received limited attention from IADL instrument developers [114]. These limitations currently hamper the adequate measurement of IADL. Most IADL questionnaires, in particular the informant-based questionnaires, pose a low level of burden to individuals with dementia and their relatives and are therefore highly feasible. Performance-based IADL instruments, on the other hand, might be more burdensome due to time requirements.

The Lawton IADL scale [115] is one of the most used and referenced traditional tools to assess instrumental abilities in the elderly such as financial management and telephone use. Although a good reliability for this scale was reported, with Cronbach’s alpha up to 0.94 [116], it suffers from several of the limitations reported above. Innovation of IADL was addressed in the development of the Amsterdam IADL questionnaire [117]. It consists of 70 activities, based on input from professionals, researchers, and caregivers, ensuring face and content validity. Modern psychometric approaches (item response theory) are applied for the scoring of the questionnaire, enabling scoring of different activities for different individuals on the same metric. In a longitudinal construct validation study, the Amsterdam IADL questionnaire was found to be sensitive to changes over time, supporting its usefulness for longitudinal studies [118].

##### Recommendations

Quality limitations hamper functional assessment. Although further research is needed to investigate quality aspects of IADL instruments, promising results have been found for several questionnaires, including the Everyday Cognition (ECog) [119], and the Cognitive Function Instrument (CFI) [120], and the Amsterdam IADL questionnaire. Therefore, the use of these tools is recommended for research purposes.

## General discussion

One main point highlighted by the project is the heterogeneity of the tests used for the assessment of the manifold aspects of clinical manifestations of dementing conditions. Various factors account for this heterogeneity, such as the preference for home-made tests, the weight of local traditions, and the context of assessment (e.g. GP practice, memory clinic, specialist clinic, or a research centre) that is related to the use of different tools according to the specific aim of assessment. In this regard, one important related issue is the “overall data fragmentation” that arises from the use of tests with different psychometric properties. This does not permit the merging of data from different centres and it makes it difficult to compare published data.

The latter observation is directly related to the second main issue of the project, i.e. the difficulty to individualise tools with adequate levels of information about psychometric properties. In the case of AD, for example, Logie et al. [121] recently emphasised that the ideal specific cognitive marker should not be affected by healthy ageing, literacy level, test/re-test, and floor effects. Moreover, it should be sensitive and specific to very early AD manifestations, be useable in primary care, non-invasive, quick to administer, and sensitive to daily living impairments. Similar considerations may be applied to other NDD.

Indeed, the working group evidenced a general issue that, across the different individual dimensions examined here, refers to the importance of paying attention to some crucial psychometric properties that neuropsychological tests should ideally fulfil. As shown in Table 2, one main point is represented by validity (i.e. “does a test measure what it is intended for?”). Content and construct validity should be well established. Moreover, test administration should follow procedures that were adopted in collecting normative data (e.g. tests for which standardisation was individually achieved are often administered within extensive neuropsychological batteries). Reliability (test/re-test and inter-rater reliability) and feasibility are other crucial prerequisites for test administration. Indeed, although, for instance, the invasiveness of the test (in terms of the discomfort caused to the patient) could affect patient performance, the issue of feasibility is often underestimated.

**Table 2:** Recommendations for test development

Psychometric properties		
Validity (i.e. does the task measure what it is intended to measure?)	Content validity Construct validity Sensitivity and specificity (according to the examination issue) Predictive validity Cross-cultural validity (including multiple language versions for verbal tests)	Clinical relevance Definitions updating according to most recent neuroscientific evidence Highly sensitive to early phases of dementia; differential diagnosis among dementias Change monitoring over time Minimal effects of literacy
Reliability	Test/re-test stability; inter-rater reliability	Avoid practice effects; parallel forms; avoid floor- and ceiling effects
Feasibility	Balance between economic and human resources costs. Availability of normative data across European countries	Optimise administration time; minimise discomfort to the patient

A related crucial issue is represented by the absence of good quality normative data (i.e. appropriate age groups and education levels) for many neuropsychological tools. In view of socio-cultural changes that occur in populations over time, a related issue refers to the need to periodically update the normative data of psychological tests. Moreover, many of the tests used in clinical settings were developed several decades ago, often in fields other than NDD (developmental disorders or stroke). There is a danger of over-simplification in applying this knowledge to NDD, where patterns of neuronal loss are often incompletely understood and by far more complex than the focal lesion models. These observations suggest particular caution in comparing data from studies using different tools with relatively dated normative references.

In conclusion, it is at the moment impossible to individualise reliable and feasible protocols and tools common to European research centres working in the field of NDD. The panel recommended psychometric tests that should be used with the aim of harmonising neuropsychological research (Table 1) according to a two-fold strategy. A first step to be followed is the achievement of a minimal data set on the proposed tools in order to reach two milestones: updated normative data and updated validity data. A second step could be the building of a common database by sharing collected data following a “big data approach”. In order to link data from different cohorts, *z* scores for every test should be made available. For instance, to improve research in multicentre studies, *z* scores based on normative data of cognitively normal subjects could be calculated [122]. These *z* scores could be pooled across different cognitive measures also allowing the computation of related composite scores. In this regard, the working group agrees on the opportunity to correctly identify tests taking into account a two-step model. First, tests to be applied for short global screening of cognitive abilities that are used to achieve general evaluation on the subject’s mental functioning, allowing the diagnosis of cognitive impairment. Second, tests with high predictive value should be recommended for the diagnosis in the very early stages of NDD, and a set of tests to be used for the purpose of differential diagnosis through the assessment of the neuropsychological profile needs to be defined.

## Conclusions

This project allowed us to identify some key points to improve clinical research in the field of dementias. Sets of indications are given to harmonise tools and procedures to achieve more reliable data on the cognitive-behavioural examination. In this regard, the results of this study should be considered as a starting point to enhance a common view and practice on NDD assessment across European countries.

As it is clear from the above discussion, the possibility to provide firm recommendations to improve neuropsychological research in dementia syndromes suffers from the current limitations of the tools and procedures available. Nevertheless, an agreement was achieved on a list of the most adequate tools and procedures to be used for each target area according to the available evidence (see Table 1). Here, we also identified un-met needs for each specific domain (see Table 1) and practical requirements of the current methods and tools of assessment, based on existing evidence and project participants’ expertise and clinical practice. In this regard, the consortium points out one clear indication on the usefulness to focus further research on those instruments that reveal potentially adequate psychometric properties for the early assessment and differential diagnosis in NDD.

The enormous progress of cognitive neurosciences in the last decades, resulting in novel, translational relevant information about the organisation of cognitive functions in the normal brain, has been exploited only to a very limited extent in proposing new measures with increased sensitivity and specificity to early stages of neurodegenerative diseases. In this vein, the need for harmonisation of tools and procedures should not hamper the possibility to implement innovative ideas. New proposals should be shared among the scientific community to explore the possibility of large-scale validation and standardisation studies at the European level.

## Abbreviations

AAT: Aachener Aphasie Test
ACE-R: Addenbrooke’s Cognitive Examination
AD: Alzheimer’s disease
ADAS-CoG: Alzheimer’s Disease Assessment Scale-Cognition
ADL: Basic activities of daily living
ALS: Amyotrophic lateral sclerosis
ANELT: Amsterdam-Nijmegen Everyday Language Test
BADL: basic activities of daily living
BADS: Behavioral Assessment of Dysexecutive Syndrome
BDAE: Boston Diagnostic Aphasia Examination
BORB: Birmingham Object Recognition Battery
BPSD: Behavioural and Psychological Symptoms of Dementia
CAMCOG-R: Cambridge Cognitive Assessment-Revised
CBD: Corticobasal degeneration
CFI: Cognitive Function Instrument
DATE: Dementia Apraxia Test
DLB: Dementia with Lewy bodies
ECogn: Everyday Cognition
EMAS: Edinburgh Motor Assessment
FAB: Frontal Assessment Battery
FBI: Frontal Behavioural Inventory
FCSRT: Free and Cued Selective Reminding Test
FTD: Frontotemporal dementia
GDS-15: Geriatric Depression Scale
HD: Huntington disease
IADL: Instrumental activities of daily living
INECO: Institute of Cognitive Neurology
ISDC: Inventaire du Syndrome Dysexécutif Comportemental
JPND: Joint Program for Neurodegenerative Diseases
MCI: Mild cognitive impairment
MDRS: Mattis Dementia Rating Scale
MMSE: Mini Mental State Examination
MoCA: Montreal Cognitive Assessment
MSA: Multiple system atrophy
NART: National Adult Reading Test
NAT: Northwestern Anagram Test
NDD: Neurodegenerative dementias
NPI: Neuropsychiatric Inventory
NPI-C: Neuropsychiatric Inventory-Clinician
NPS: Neuropsychiatric symptoms
PCA: Posterior cortical atrophy
PD: Parkinson’s disease
PDD: Parkinson’s disease dementia
PPA: Primary progressive aphasia
PSP: Supranuclear palsy
PSPRS: Progressive Supranuclear Palsy Rating Scale
SEA: Social and Emotional Assessment battery
SYDBAT: Sydney Language Battery
ToM: Theory of mind
TROG: Test for the reception of grammar
UHDRS: Unified Huntington’s Disease Rating Scale
UMSARS: Unified Multiple System Atrophy Rating Scale
UPDRS: Unified Parkinson’s Disease Rating Scale
VaD: Vascular dementia
VOSP: Visual Object and Space Perception battery
WAB: Western Aphasia Battery
WG: Working Group

## References

[1] Daffner KR, Gale SA, Barrett AM, Boeve BF, Chatterjee A, Coslett HB (2015). Improving clinical cognitive testing: report of the AAN Behavioral Neurology Section Workgroup. Neurology.

[2] Wilcock GK, Hope RA, Brooks DN, Lantos PL, Oppenheimer C, Reynolds GP (1989). Recommended minimum data to be collected in research studies on Alzheimer’s disease. The MRC (UK) Alzheimer’s Disease Workshop Steering Committee. J Neurol Neurosurg Psychiatry.

[3] Hachinski V, Iadecola C, Petersen RC, Breteler MM, Nyenhuis DL, Black SE (2006). National Institute of Neurological Disorders and Stroke-Canadian Stroke Network vascular cognitive impairment harmonization standards. Stroke.

[4] Diaz PR, Gregório GP, Ribera Casado M, Reynish E, Ousset JP, Vellas B (2005). The need for a consensus in the use of assessment tools for Alzheimer’s disease: the Feasibility Study (assessment tools for dementia in Alzheimer Centres across Europe), a European Alzheimer’s Disease Consortium’s (EADC) survey. Int J Geriatr Psychiatry.

[5] Maruta C, Guerreiro M, de Mendonça A, Hort J, Scheltens P (2011). The use of neuropsychological tests across Europe: the need for a consensus in the use of assessment tools for dementia. Eur J Neurol.

[6] Dubois B, Feldman HH, Jacova C, Hampel H, Molinuevo JL, Blennow K (2014). Advancing research diagnostic criteria for Alzheimer’s disease: the IWG-2 criteria. Lancet Neurol.

[7] McKhann GM, Knopman DS, Chertkow H, Hyman BT, Jack CR, Kawas CH (2011). The diagnosis of dementia due to Alzheimer’s disease: recommendations from the National Institute on Aging-Alzheimer’s Association workgroups on diagnostic guidelines for Alzheimer’s disease. Alzheimer’s Dement.

[8] Crutch SJ, Schott JM, Rabinovici GD, Boeve BF, Cappa SF, Dickerson BC (2013). Shining a light on posterior cortical atrophy. Alzheimers Dement.

[9] Rascovsky K, Hodges JR, Knopman D, Mendez MF, Kramer JH, Neuhaus J (2011). Sensitivity of revised diagnostic criteria for the behavioural variant of frontotemporal dementia. Brain.

[10] Gorno-Tempini ML, Hillis AE, Weintraub S, Kertesz A, Mendez M, Cappa SF (2011). Classification of primary progressive aphasia and its variants. Neurology.

[11] Folstein MF, Folstein SE, McHugh PR (1975). “Mini Mental State” a practical method for grading the cognitive state of patients for the clinicians. J Psychiat Res.

[12] Marioni RE, Chatfield M, Brayne C, Matthews FE, Medical Research Council Cognitive Function and Ageing Study Group (2011). The reliability of assigning individuals to cognitive states using the Mini Mental-State Examination: a population-based prospective cohort study. BMC Med Res Methodol.

[13] Mitolo M, Salmon DP, Gardini S, Galasko D, Grossi E, Caffarra P (2014). The New Qualitative Scoring MMSE Pentagon Test (QSPT) as a valid screening tool between autopsy-confirmed dementia with Lewy bodies and Alzheimer’s disease. J Alzheimers Dis.

[14] Nieuwenhuis-Mark RE (2010). The death knoll for the MMSE: has it outlived its purpose?. J Geriatr Psychiatry Neurol.

[15] Nasreddine ZS, Phillips NA, Bédirian V, Charbonneau S, Whitehead V, Collin I (2005). The Montreal Cognitive Assessment, MoCA: a brief screening tool for mild cognitive impairment. J Am Geriatr Soc.

[16] Freitas S, Simões MR, Alves L, Santana I (2013). Montreal cognitive assessment: validation study for mild cognitive impairment and Alzheimer disease. Alzheimer Dis Assoc Disord.

[17] Dubois B, Slachevsky A, Litvan I, Pillon BF (2000). The FAB—a frontal assessment battery at bedside. Neurology.

[18] Castiglioni S, Pelati O, Zuffi M, Somalvico F, Marino L, Tentorio T (2006). The frontal assessment battery does not differentiate frontotemporal dementia from Alzheimer’s disease. Dement Geriatr Cogn Disord.

[19] Lipton AM, Ohman KA, Womack KB, Hynan LS, Ninman ET, Lacritz LH (2005). Subscores of the FAB differentiate frontotemporal lobar degeneration from AD. Neurology.

[20] Doraiswamy PM, Krishen A, Stallone F, Martin WL, Potts NL, Metz A (1995). Cognitive performance on the Alzheimer’s Disease Assessment Scale: effect of education. Neurology.

[21] Verhey FR, Huppert FA, Korten EC, Houx P, de Vugt M, van Lang N (2003). Cross-national comparisons of the Cambridge Cognitive Examination-revised: the CAMCOG-R: results from the European Harmonization Project for Instruments in Dementia. Age Ageing.

[22] Mioshi E, Dawson K, Mitchell J, Arnold R, Hodges JR (2006). The Addenbrooke’s Cognitive Examination Revised (ACE‐R): a brief cognitive test battery for dementia screening. Int J Geriatr Psychiatry.

[23] Rittman T, Ghosh BC, McColgan P, Breen DP, Evans J, Williams-Gray CH (2013). The Addenbrooke’s Cognitive Examination for the differential diagnosis and longitudinal assessment of patients with parkinsonian disorders. J Neurol Neurosurg Psychiatry.

[24] Schmidt R, Freidl W, Fazekas F, Reinhart B, Grieshofer P, Koch M (1994). The Mattis Dementia Rating Scale. Normative data from 1,001 healthy volunteers. Neurology.

[25] Pirogovsky E, Schiehser DM, Litvan I, Obtera KM, Burke MM, Lessig SL (2014). The utility of the Mattis Dementia Rating Scale in Parkinson’s disease mild cognitive impairment. Parkinsonism Relat Disord.

[26] Borson S, Scanlan J, Brush M, Vitaliano P, Dokmak A (2000). The mini-cog: a cognitive “vital signs” measure for dementia screening in multi-lingual elderly. Int J Geriatr Psychiatry.

[27] Lowndes GJ, Saling MM, Ames D, Chiu E, Gonzalez LM, Savage GR (2008). Recall and recognition of verbal paired associates in early Alzheimer’s disease. J Int Neuropsychol Soc.

[28] Savage RM, Gouvier WD (1992). Rey Auditory-Verbal Learning Test: the effects of age and gender, and norms for delayed recall and story recognition trials. Arch Clin Neuropsychol.

[29] Dubois B, Touchon J, Portet F, Ousset PJ, Vellas B, Michel B (2002). “The 5 words”: a simple and sensitive test for the diagnosis of Alzheimer’s disease. Presse Med.

[30] Grober E, Sanders AE, Hall C, Lipton RB (2010). Free and cued selective reminding identifies very mild dementia in primary care. Alzheimer Dis Assoc Disord.

[31] Wagner M, Wolf S, Reischies FM, Daerr M, Wolfsgruber S, Jessen F (2012). Biomarker validation of a cued recall memory deficit in prodromal Alzheimer disease. Neurology.

[32] Barbeau E, Didic M, Tramoni E, Felician O, Joubert S, Sontheimer A (2004). Evaluation of visual recognition memory in MCI patients. Neurology.

[33] Parra MA, Abrahams S, Fabi K, Logie R, Luzzi S, Della SS (2009). Short-term memory binding deficits in Alzheimer’s disease. Brain.

[34] Della Sala S, Parra MA, Fabi K, Luzzi S, Abrahams S (2012). Short-term memory binding is impaired in AD but not in non-AD dementias. Neuropsychologia.

[35] Papageorgiou SG, Economou A, Routsis C (2014). The 5 Objects Test: a novel, minimal-language, memory screening test. J Neurol.

[36] Costa A, Fadda L, Perri R, Brisindi M, Lombardi MG, Caltagirone C (2015). Sensitivity of a time-based prospective memory procedure in the assessment of amnestic mild cognitive impairment. J Alzheimers Dis.

[37] Masur DM, Sliwinski M, Lipton RB, Blau AD, Crystal HA (1994). Neuropsychological prediction of dementia and the absence of dementia in healthy elderly persons. Neurology.

[38] Locascio JJ, Growdon JH, Corkin S (1995). Cognitive test performance in detecting, staging, and tracking Alzheimer’s disease. Arch Neurol.

[39] Kaplan E, Goodglass H, Weintraub S (1983). Boston Naming Test.

[40] Katsumata Y, Mathews M, Abner EL, Jicha GA, Caban-Holt A, Smith CD (2015). Assessing the discriminant ability, reliability, and comparability of multiple short forms of the Boston Naming Test in an Alzheimer’s disease center cohort. Dement Geriatr Cogn Disord.

[41] McKenna P, Warrington EK (1980). Testing for nominal dysphasia. J Neurol Neurosurg Psychiatry.

[42] Huber W. Aachener aphasie test (AAT). Verlag für Psychologie: Dr. CJ Hogrefe: 1983

[43] De Jager CA, Hogervorst E, Combrinck M, Budge MM (2003). Sensitivity and specificity of neuropsychological tests for mild cognitive impairment, vascular cognitive impairment and Alzheimer’s disease. Psychol Med.

[44] Brambati SM, Myers D, Wilson A, Rankin KP, Allison SC, Rosen HJ (2006). The anatomy of category-specific object naming in neurodegenerative diseases. J Cogn Neurosci.

[45] Stuss DT, Alexander MP, Hamer L, Palumbo C, Dempster R, Binns M (1998). The effects of focal anterior and posterior brain lesions on verbal fluency. J Int Neuropsychol Soc.

[46] Howard D, Patterson KE. The Pyramids and Palm Trees Test: a test of semantic access from words and pictures. England: Thames Valley Test Company; 1992.

[47] Bozeat S, Lambon Ralph MA, Patterson K, Garrard P, Hodges JR (2000). Non-verbal semantic impairment in semantic dementia. Neuropsychologia.

[48] Wilson SM, Henry ML, Besbris M, Ogar JM, Dronkers NF, Jarrold W (2010). Connected speech production in three variants of primary progressive aphasia. Brain.

[49] Weintraub S, Mesulam MM, Wieneke C, Rademaker A, Rogalski EJ, Thompson CK (2009). The northwestern anagram test: measuring sentence production in primary progressive aphasia. Am J Alzheimers Dis Other Demen.

[50] De Renzi E, Vignolo LA (1962). The Token Test: a sensitive test to detect receptive disturbances in aphasics. Brain.

[51] Bishop DVM (1983). Comprehension of English syntax by profoundly deaf children. J Child Psychol Psychiatry.

[52] Curtiss S. Curtiss–Yamada comprehensive language evaluation. Unpublished test, Los Angeles: UCLA; 1988.

[53] Nelson HE, Willison J (1991). The Nelson Adult Reading Test (NART): test manual.

[54] Adlam AL, Patterson K, Bozeat S, Hodges JR (2010). The Cambridge Semantic Memory Test Battery: detection of semantic deficits in semantic dementia and Alzheimer’s disease. Neurocase.

[55] Savage S, Hsieh S, Leslie F, Foxe D, Piguet O, Hodges JR (2013). Distinguishing subtypes in primary progressive aphasia: application of the Sydney language battery. Dement Geriatr Cogn Dis.

[56] Blomert L, Kean M, Koster C, Schokker J (1994). Amsterdam—Nijmegen everyday language test: construction, reliability and validity. Aphasiology.

[57] Chan RC, Shum D, Toulopoulou T, Chen EY (2008). Assessment of executive functions: review of instruments and identification of critical issues. Arch Clin Neuropsychol.

[58] Torralva T, Cuitino MM, Manes F, Dickerson BC (2016). Neuropsychological assessment of frontotemporal dementia. Hodges’ frontotemporal dementia.

[59] Stopford CL, Thompson JC, Neary D, Richardson AMT, Snowden JS (2012). Working memory, attention and executive function in Alzheimer’s disease and frontotemporal dementia. Cortex.

[60] Collette F, Schmidt C, Scherrer C, Adam S, Salmon E (2009). Specificity of inhibitory deficits in normal aging and Alzheimer’s disease. Neurobiol Aging.

[61] Collette F, Amieva H, Adam S, Hogge M, Van der Linden M, Fabrigoule C (2007). Comparison of inhibitory functioning in mild Alzheimer’s disease and frontotemporal dementia. Cortex.

[62] Lagarde J, Valabrègue R, Corvol JC, Garcin B, Volle E, Le Ber I (2015). Why do patients with neurodegenerative frontal syndrome fail to answer: ‘in what way are an orange and a banana alike?’. Brain.

[63] Burgess PW, Alderman N, Evans J, Emslie H, Wilson BA (1998). The ecological validity of tests of executive functions. J Int Neuropsychol Soc.

[64] Torralva T, Roca M, Gleichgerrcht E, Bekinschtein T, Manes F (2009). A neuropsychological battery to detect specific executive and social cognitive impairments in early frontotemporal dementia. Brain.

[65] Godefroy O, GREFEX (2008). Fonctions exécutives et pathologies neurologiques et psychiatriques. Evaluation en pratique clinique.

[66] Wilson BA, Evans JJ, Emslie H, Alderman N, Burgess P (1998). The development of an ecologically valid test for assessing patients with a dysexecutive syndrome. Neuropsychol Rehabil.

[67] Ekman P, Friesen WV, Ellsworth P (1972). Emotion in the human face.

[68] Bechara A, Tranel D, Damasio H (2000). Characterization of the decision-making deficit of patients with ventromedial prefrontal cortex lesions. Brain.

[69] Bertoux M, Funkiewiez A, O’Callaghan C, Dubois B, Hornberger M (2013). Sensitivity and specificity of ventromedial prefrontal cortex tests in behavioral variant frontotemporal dementia. Alzheimer’s Dement.

[70] Bertoux M, Volle E, de Souza LC, Funkiewiez A, Dubois B, Habert MO (2014). Neural correlates of the mini-SEA (Social cognition and Emotional Assessment) in behavioral variant frontotemporal dementia. Brain Imaging Behav.

[71] Hutchison KA, Balota DA, Duchek JM (2010). The utility of Stroop Task switching as a marker for early stage Alzheimer’s disease. Psychol Aging.

[72] Reitan R (1970). Trail-making test.

[73] Heaton RK, Chelune GJ, Talley JL, Kay GG, Curtiss G (1993). Wisconsin Card Sorting Test Manual: revised and expanded.

[74] Henry JD, Crawford JR, Phillips LH (2004). Verbal fluency performance in dementia of the Alzheimer’s type: a meta-analysis. Neuropsychologia.

[75] Shakespeare TJ, Yong KX, Foxe D, Hodges J, Crutch SJ (2015). Pronounced impairment of everyday skills and self-care in posterior cortical atrophy. J Alzheimers Dis.

[76] Goodale MA, Milner AD (1992). Separate visual pathways for perception and action. Trends Neurosci.

[77] Lehmann M, Barnes J, Ridgway GR, Wattam-Bell J, Warrington EK, Fox NC, et al. Basic visual function and cortical thickness patterns in posterior cortical atrophy. Cereb Cortex. 2011;212122-32. doi: 10.1093/cercor/bhq287 10.1093/cercor/bhq287 21310781

[78] Twum M, Parenté R (1994). Role of imagery and verbal labeling in the performance of paired associates tasks by persons with closed head injury. J Clin Exp Neuropsychol.

[79] Warrington EK (1996). Studies of retrograde memory: a long-term view. Proc Natl Acad Sci U S A.

[80] McKeith IG, Dickson DW, Lowe J, Emre M, O’Brien JT, Feldman H (2005). Diagnosis and management of dementia with Lewy bodies: third report of the DLB Consortium. Neurology.

[81] Mathew R, Bak TH, Hodges JR (2012). Diagnostic criteria for corticobasal syndrome: a comparative study. J Neurol Neurosurg Psychiatry.

[82] Crutch SJ, Lehmann M, Schott JM, Rabinovici GD, Rossor MN, Fox NC (2012). Posterior cortical atrophy. Lancet Neurol.

[83] Warrington EK, James M (1991). A new test of object decision: 2D silhouettes featuring a minimal view. Cortex.

[84] Quental NB, Brucki SM, Bueno OF (2013). Visuospatial function in early Alzheimer’s disease—the use of the Visual Object and Space Perception (VOSP) battery. PLoS One.

[85] Riddoch MJ, Humphreys GW (1993). The Birmingham Object Recognition Battery (BORB).

[86] Benton AL, Varney NR, Hamsher KS (1978). Visuospatial judgment: a clinical test. Arch Neurol.

[87] Simard M, van Reekum R, Myran D (2003). Visuospatial impairment in dementia with Lewy bodies and Alzheimer’s disease: a process analysis approach. Int J Geriatr Psychiatry.

[88] Osterrieth PA (1944). Le test de copie d’une figure complex: contribution à l’étude de la perception et de la memoir. Arch Psychol.

[89] Siri S, Benaglio I, Frigerio A, Binetti G, Cappa SF (2001). A brief neuropsychological assessment for the differential diagnosis between frontotemporal dementiaand Alzheimer’s disease. Eur J Neurol.

[90] Possin KL, Laluz VR, Alcantar OZ, Miller BL, Kramer JH (2011). Distinct neuroanatomical substrates and cognitive mechanisms of figure copy performance in Alzheimer’s disease and behavioral variant frontotemporal dementia. Neuropsychologia.

[91] Cerejeira J, Lagarto L, Mukaetova-Ladinska EB (2012). Behavioral and psychological symptoms of dementia. Front Neurol.

[92] Monastero R, Mangialasche F, Camarda C, Ercolani S, Camarda R (2009). A systematic review of neuropsychiatric symptoms in mild cognitive impairment. J Alzheimers Dis.

[93] Spalletta G, Musicco M, Padovani A, Rozzini L, Perri R, Fadda L (2010). Neuropsychiatric symptoms and syndromes in a large cohort of newly diagnosed, untreated patients with Alzheimer. Am J Geriatr Psychiatry.

[94] Kertesz A, Davidson W, Fox H (1997). Frontal Behavioral Inventory: diagnostic criteria for frontal lobe dementia. Can J Neurol Sci.

[95] Reisberg B, Borenstein J, Salob SP, Ferris SH, Franssen E, Georgotas A (1987). Behavioral symptoms in Alzheimer’s disease: phenomenology and treatment. J Clin Psychiatry.

[96] Cummings JL (1997). The Neuropsychiatric Inventory: assessing psychopathology in dementia patients. Neurology.

[97] Suárez-González A, Serrano-Pozo A, Arroyo-Anlló EM, Franco-Macías E, Polo J, García-Solís D (2014). Utility of neuropsychiatric tools in the differential diagnosis of dementia with Lewy bodies and Alzheimer’s disease: quantitative and qualitative findings. Int Psychogeriatr.

[98] Rosenberg PB, Drye LT, Porsteinsson AP, Pollock BG, Devanand DP, Frangakis C (2015). Change in agitation in Alzheimer’s disease in the placebo arm of a nine-week controlled trial. Int Psychogeriatr.

[99] Atri A, Hendrix SB, Pejović V, Hofbauer RK, Edwards J, Molinuevo JL, Graham SM (2015). Cumulative, additive benefits of memantine-donepezil combination over component monotherapies in moderate to severe Alzheimer’s dementia: a pooled area under the curve analysis. Alzheimers Res Ther.

[100] De Medeiros K, Robert P, Gauthier S, Stella F, Politis A, Leoutsakos J (2010). The Neuropsychiatric Inventory-Clinician rating scale (NPI-C): reliability and validity of a revised assessment of neuropsychiatric symptoms in dementia. Int Psychogeriatr.

[101] Radakovic R, Abrahams S (2014). Developing a new apathy measurement scale: Dimensional Apathy Scale. Psychiatry Res.

[102] Yesavage JA, Brink TL, Rose TL, Lum O, Huang V, Adey MB (1983). Development and validation of a geriatric depression screening scale: a preliminary report. J Psychiat Res.

[103] Lesourd M, Le Gall D, Baumard J, Croisile B, Jarry C, Osiurak F (2013). Apraxia and Alzheimer’s disease: review and perspectives. Neuropsychol Rev.

[104] Scarmeas N, Hadjigeorgiou GM, Papadimitriou A, Dubois B, Sarazin M, Brandt J (2004). Motor signs during the course of Alzheimer disease. Neurology.

[105] Ryan NS, Shakespeare TJ, Lehmann M, Keihaninejad S, Nicholas JM, Leung KK (2014). Motor features in posterior cortical atrophy and their imaging correlates. Neurobiol Aging.

[106] Johnen A, Frommeyer J, Modes F, Wiendl H, Duning T, Lohmann H (2015). Dementia Apraxia Test (DATE): a brief tool to differentiate behavioral variant frontotemporal dementia from Alzheimer’s. J Alzheimers Dis.

[107] Bak T, Bennett G, Symonds A, PAI S (2015). Motor symptoms in healthy ageing and dementia: frequency, patterns and the relation between motor and cognitive functions. Eur J Neurol.

[108] Petersen R, Doody R, Kurz A, Mohs R, Morris J, Rabins P (2001). Current concepts in mild cognitive impairment. Arch Neurol.

[109] Sikkes SAM, Visser PJ, Knol DL, de Lange-de Klerk ESM, Tsolaki M, Frisoni GB (2011). Do instrumental activities of daily living predict dementia at 1- and 2-year follow-up? Findings from the Development of Screening guidelines and diagnostic Criteria for Predementia Alzheimer’s disease study. J Am Geriatr Soc.

[110] Jekel K, Damian M, Wattmo C, Hausner L, Bullock R, Connelly PJ (2015). Mild cognitive impairment and deficits in instrumental activities of daily living: a systematic review. Alzheimers Res Ther.

[111] Desai AK, Grossberg GT, Sheth DN (2004). Activities of daily living in patients with dementia: clinical relevance, methods of assessment and effects of treatment. CNS Drugs.

[112] Sikkes SAM, de Lange-de Klerk ESM, Pijnenburg YAL, Scheltens P, Uitdehaag BMJ (2009). A systematic review of Instrumental Activities of Daily Living scales in dementia: room for improvement. J Neurol Neurosurg Psychiatry.

[113] Gold DA (2012). An examination of instrumental activities of daily living assessment in older adults and mild cognitive impairment. J Clin Exp Neuropsychol.

[114] Marshall G, Amariglio RE, Sperling R, Rentz DM (2012). Activities of daily living: where do they fit in the diagnosis of Alzheimer’s disease?. Neurodegener Dis Manag.

[115] Lawton MP, Brody EM (1969). Assessment of older people: self-maintaining and instrumental activities of daily living. Gerontologist.

[116] Vergara I, Bilbao A, Orive M, Garcia-Gutierrez S, Navarro G, Quintana JM (2012). Validation of the Spanish version of the Lawton IADL Scale for its application in elderly people. Health Qual Life Outcomes.

[117] Sikkes SAM, Pijnenburg YAL, Knol DL, de Lange-de Klerk ESM, Scheltens P, Uitdehaag BMJ (2013). Assessment of instrumental activities of daily living in dementia: diagnostic value of the Amsterdam Instrumental Activities of Daily Living Questionnaire. J Geriatr Psychiatry Neurol.

[118] Koster N, Knol DL, Uitdehaag BMJ, Scheltens P, Sikkes SAM (2015). The sensitivity to change over time of the Amsterdam IADL Questionnaire. Alzheimer’s Dement.

[119] Farias ST, Mungas D, Reed BR, Cahn-Weiner D, Jagust W, Baynes K (2008). The measurement of everyday cognition (ECog): scale development and psychometric properties. Neuropsychology.

[120] Walsh SP, Raman R, Jones KB, Aisen PS (2006). ADCS Prevention Instrument Project: the Mail-In Cognitive Function Screening Instrument (MCFSI). Alzheimer Dis Assoc Disord.

[121] Logie R, Parra Rodriguez M, Della SS (2015). From cognitive science to dementia assessment. Behav Brain Sc.

[122] Visser PJ, Verhey FR, Boada M, Bullock R, De Deyn PP, Frisoni GB (2008). Development of screening guidelines and clinical criteria for predementia Alzheimer’s disease. The DESCRIPA Study. Neuroepidemiology.

